# High rate of adaptation of mammalian proteins that interact with *Plasmodium* and related parasites

**DOI:** 10.1371/journal.pgen.1007023

**Published:** 2017-09-28

**Authors:** Emily R. Ebel, Natalie Telis, Sandeep Venkataram, Dmitri A. Petrov, David Enard

**Affiliations:** 1 Department of Biology, Stanford University, Stanford, California, United States of America; 2 Program in Biomedical Informatics, Stanford University, Stanford, California, United States of America; The Pennsylvania State University, UNITED STATES

## Abstract

*Plasmodium* parasites, along with their Piroplasm relatives, have caused malaria-like illnesses in terrestrial mammals for millions of years. Several *Plasmodium*-protective alleles have recently evolved in human populations, but little is known about host adaptation to blood parasites over deeper evolutionary timescales. In this work, we analyze mammalian adaptation in ~500 *Plasmodium*- or Piroplasm- interacting proteins (PPIPs) manually curated from the scientific literature. We show that (i) PPIPs are enriched for both immune functions and pleiotropy with other pathogens, and (ii) the rate of adaptation across mammals is significantly elevated in PPIPs, compared to carefully matched control proteins. PPIPs with high pathogen pleiotropy show the strongest signatures of adaptation, but this pattern is fully explained by their immune enrichment. Several pieces of evidence suggest that blood parasites specifically have imposed selection on PPIPs. First, even non-immune PPIPs that lack interactions with other pathogens have adapted at twice the rate of matched controls. Second, PPIP adaptation is linked to high expression in the liver, a critical organ in the parasite life cycle. Finally, our detailed investigation of alpha-spectrin, a major red blood cell membrane protein, shows that domains with particularly high rates of adaptation are those known to interact specifically with *P*. *falciparum*. Overall, we show that host proteins that interact with *Plasmodium* and Piroplasm parasites have experienced elevated rates of adaptation across mammals, and provide evidence that some of this adaptation has likely been driven by blood parasites.

## Author summary

Malaria caused by the parasite *Plasmodium falciparum* remains the third-most deadly infectious disease of humans. Over the last 75,000 years, partial genetic resistance to malaria has evolved several times, earning malaria the title of "one of the strongest selective forces on the human genome." Yet, these human adaptations are only the most recent maneuvers in an ancient evolutionary war between host and parasite. Relatives of *Plasmodium* infect a variety of mammalian species today, and these large groups of hosts and parasites have likely coevolved for over 100 million years. Here, we identify 490 host genes that have been experimentally linked to the outcome of parasite infection in mammals, representing approximately 5% of all conserved mammalian proteins. In many cases, these proteins have also been linked to viral or bacterial infections. We show that parasite-interacting proteins have experienced ~3 times more adaptive substitutions than expected over mammalian evolution, and that blood parasites have left their own significant mark on our ancestors' genomes. We also identify one target of long-term adaptation to *Plasmodium—*the red blood cell protein alpha-spectrin—that may be involved in human susceptibility to malaria, demonstrating the value of considering modern disease in its long-term evolutionary context.

## Introduction

**Fig 1:**
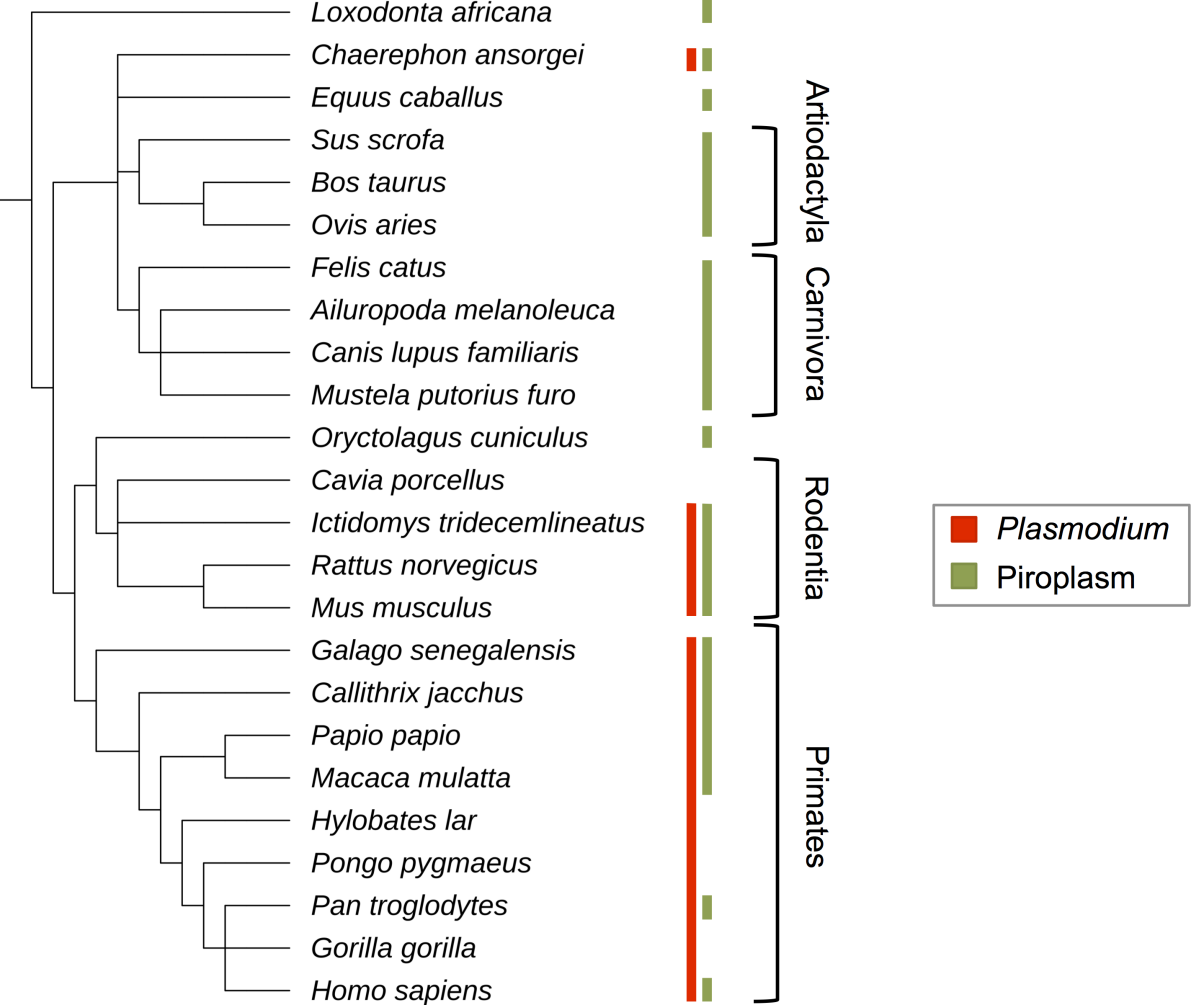
Observations of *Plasmodium* and Piroplasm infection in well-sequenced mammal groups. References for parasite infection in these species or their close relatives are given in S1 Table.

Malaria is one of the world's most notorious infectious diseases, responsible for billions of illnesses and millions of deaths in the last fifty years alone [1]. Human malaria is caused by five species in the genus *Plasmodium*, which are evolutionarily related to *Babesia*, *Theileria*, and other parasites in the order Piroplasmida. Approximately fifty *Plasmodium* species cause malaria in primates, rodents, and bats [2], while Piroplasms infect a wider range of mammals (Fig 1). Although some wild animals appear to host malaria parasites without ill effects (e.g. [3]), others are known to suffer serious symptoms and death, especially when exposed to novel parasites [4, 5]. The severity of infection may then depend on the population history of exposure and individual acquired immunity, as it does in humans [6].

Parasites and other pathogens are important drivers of adaptive evolution in their hosts [7]. In the specific case of humans and *Plasmodium*, genetic variation in about 35 red blood cell or immune proteins has been associated with protection from severe complications of malaria, if not outright resistance (reviewed in [8–10]). Some of these protective genes, including HBB, DARC, and GYPA, have been supported by population genetic evidence of selection in African or Southeast Asian populations within the last 75,000 years (e.g. [11–13]). Malaria has consequently been labeled "one of the strongest selective forces on the human genome" [9, 10], though this statement has never been quantified.

Human adaptation to malaria is likely occurring within the broader context of mammalian adaptation to widespread blood parasites. The common ancestor of modern humans existed perhaps 200,000 years ago, while the common ancestor of placental mammals dates back ~105 million years [14, 15]. For comparison, the parasite genus *Plasmodium* experienced a major radiation ~129 million years ago [16]. *Plasmodium* and Piroplasms have likely infected mammals for as long as mammals have existed, but the evolutionary consequences of this long-standing relationship have never been investigated.

Despite their age and diversity, *Plasmodium* and Piroplasms cause disease through similar mechanisms, including transmission to and from mammalian blood by the bite of a mosquito or tick. *Plasmodium* cells migrate first to the liver, multiply within hepatocytes, and emerge several days later to invade red blood cells (RBCs) [17]. *Babesia* parasites invade RBCs directly, while *Theileria* parasites infect both red and white blood cells [18, 19]. Although Piroplasms like *Babesia* and *Theileria* are thought to lack a liver stage [20], their infections cause substantial liver damage through increased coagulation and other mechanisms [21–23]. Parasitized cells also adhere to capillaries lining the liver, lung, brain, and other tissues, which can impair circulation and lead to life-threatening organ dysfunction (e.g. [18, 24–26]). Finally, each parasitic infection triggers a complex immune response from the host, including the removal of infected RBCs from circulation by the spleen (e.g. [27]).

The complexity of these host-parasite interactions makes it difficult to precisely measure their evolutionary impact. One important reason is that our knowledge of host responses is biased toward convenient samples, like blood cells, from specific groups, like humans and *Plasmodium*. We particularly lack information across the extant diversity of parasite and host species [2]. A second key reason is that host genes relevant to malaria are likely to be pleiotropically involved with other selected phenotypes, including responses to other pathogens [28]. In particular, some *Plasmodium*-associated genes in humans are also associated with viruses or bacteria, making it difficult to attribute their evolution specifically to pressure from *Plasmodium* [29, 30]. Parsing the contribution of various pathogens to host evolution thus requires a broader understanding of many host genes, many tissues, and many pathogens.

In this work, we examine patterns of adaptation and functional pleiotropy in a set of ~500 *Plasmodium*- or Piroplasm-interacting proteins (PPIPs) manually curated from the literature. These PPIPs represent about 5% of the mammalian proteome, as defined by the set of 9,338 proteins conserved across 24 well-sequenced mammal species. Previously, evolutionary analysis of an externally defined gene set has proven useful for detecting polygenic adaptation [7, 31–33]. Here, because PPIPs represent a relatively small fraction of all conserved mammalian genes, we use permutation tests to compare PPIPs to a background of non-PPIP controls. That is, we compare PPIPs to many sets of other mammalian proteins, which we match to PPIPs by a number of important metrics. This approach has recently been used by [31] to identify viruses as a dominant driver of adaptation in mammals.

Overall, we demonstrate that PPIPs have experienced ~3 times more adaptive substitutions than expected throughout mammalian evolution. The strongest adaptive signals are present in PPIPs with immune functions, which are highly pleiotropic with respect to other pathogens. However, we detect a significant excess of adaptation even in non-immune PPIPs that are not known to interact with pathogens beside *Plasmodium*. Additional evidence suggests that the red blood cell protein alpha-spectrin, as well as PPIPs highly expressed in the liver, may have played key roles in adaptation to blood parasites. Overall, our work supports the hypothesis that *Plasmodium* and Piroplasm parasites—not unlike other classes of pathogens—have been important and long-standing drivers of evolutionary change in mammals.

## Results

### Identification of 490 *Plasmodium*- or Piroplasm-interacting proteins (PPIPs)

Malaria-like illnesses generate substantial health and economic burdens in humans, livestock, and pets [1, 34]. These costs have motivated a large body of research into host-parasite interactions and host responses to infection. We queried the PubMed database for scientific papers whose abstracts mentioned the name of a host gene along with the terms *malaria*, *Plasmodium*, *Babesia*, *Theileria*, *Rangelia*, or *Cytauxzoon*, the latter four being the best-studied Piroplasmid genera (Methods, PPIP Identification). To focus on mammalian evolution, we limited our search to 9,338 protein-coding genes that are conserved in 24 mammalian species with high-quality reference genomes (Fig 1; Methods, Mammalian Orthologs; [31]). Most of these mammalian species belong to one of four orders—primates, rodents, artiodactyls, or carnivores—and represent a range of susceptibilities to our focal parasites (Fig 1).

**Fig 2:**
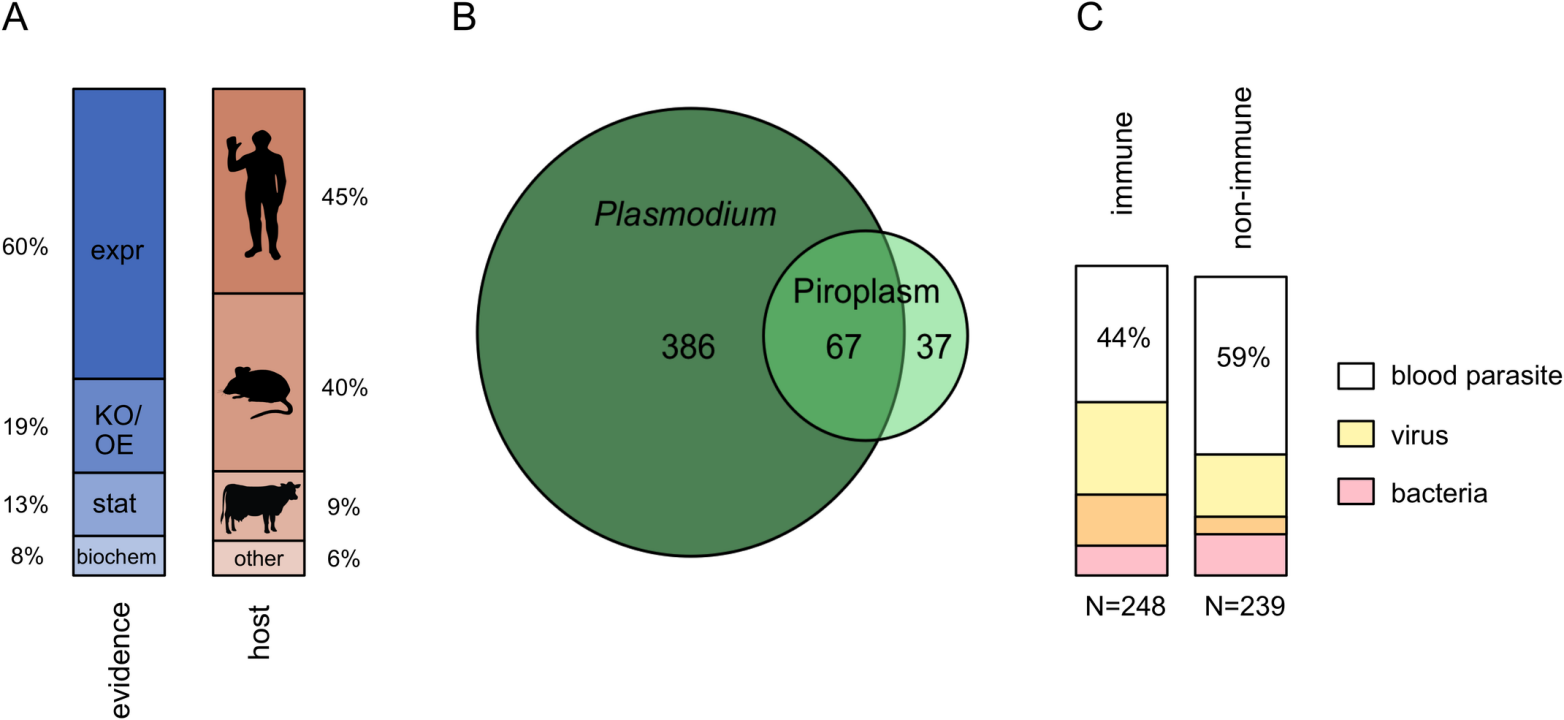
Mammalian proteins that interact with blood parasites. **(A)** Evidence types and host species from studies that identified 490 *Plasmodium*- and Piroplasm- Interacting Proteins (PPIPs). Percentages indicate the contribution to total evidence or host species. **expr**–correlational expression change; **KO/OE**–genetic knockout or overexpression experiment; **stat**–statistical association between genotype and disease severity; **biochem–**molecular interaction with a parasite protein. **(B)** Number of PPIPs identified from studies on *Plasmodium*, Piroplasms, or both. **(C)** Proportion of immune and non-immune PPIPs that also interact with viral or bacterial pathogens.

This search returned ~35,000 papers associated with ~5,000 mammalian genes. However, the vast majority of these results were false positives. Many short acronyms that identify genes have multiple meanings, and many papers containing these acronyms do not concern genes or proteins. We manually curated paper titles and abstracts to identify just 786 papers linking 490 proteins to *Plasmodium* or Piroplasms via four types of phenotypic evidence: (1) biochemical interaction between a mammalian and parasite protein; (2) statistical association between genetic variation and disease susceptibility; (3) knockout or overexpression studies impacting susceptibility; and (4) low-throughput studies showing a change in gene expression during infection (Fig 2A; S2 Table).

Nearly half of the 490 PPIPs (45%) were supported by multiple studies, and 35% by multiple sources of evidence. Expression changes were the most common form of evidence (85% of PPIPs, or 60% of total evidence), with about 43% of PPIPs identified only via expression changes. Expression-based PPIPs likely represent both direct and indirect interactions with parasites, given the size and interconnectedness of gene expression networks (e.g. [35]). We have attempted to limit indirect interactions by excluding high-throughput expression experiments, and we later show that all four evidence types, including expression changes, identify sets of genes with elevated rates of adaptation (S4 Fig). Consequently, we chose to analyze all PPIPs together without making distinctions based on evidence type.

The majority of PPIPs were linked to *Plasmodium* in studies of humans and mice (Fig 2). A fifth of PPIPs (21%) were linked to Piroplasms in studies of cows, dogs, and other mammal species (Fig 2). *Plasmodium-* and Piroplasm-interacting proteins overlap substantially, ~14 times more than expected by chance (p<1x10^-5^, Fig 2B). This overlap is consistent with the similar life cycles of *Plasmodium* and Piroplasms, as well as the conservation of host responses to these parasites across mammals.

To ask whether PPIPs generally perform functions relevant to malaria, we also tested 17,696 GO functional categories for PPIP enrichment (Methods, Protein Metrics). After correcting for multiple testing, over 1,200 categories contained significantly more PPIPs than expected (S3 Table). The most enriched categories were dominated by immune functions, with 51% of PPIPs falling under *immune system process* (p = 6.16 x 10^−94^) and 83% under *response to stimulus* (p = 7.89 x 10^−69^) (Table 1). Other highly enriched categories indicate functions more specific to malaria pathology, including *cell adhesion* (p = 9.89 x 10^−32^), *hemostasis* (p = 1.68 x 10^−35^), and *hemopoiesis* (p = 2.20 x 10^−37^) (Table 1). Together, these results reflect the expected functions of host genes ascertained through studies of malaria-relevant processes.

**Table 1:** Top 20 GO functions enriched for PPIPs.

Function	Code	N PPIPs	N expected	*p*
Immune system process	GO:0002376	248	55	6.16 × 10^−94^
Response to stimulus	GO:0050896	406	213	7.89 × 10^−69^
Cell activation	GO:0001775	138	19	2.92 × 10^−68^
Cytokine production	GO:0001816	107	11	4.66 × 10^−61^
Cell death	GO:0008219	178	52	4.43 × 10^−47^
Signaling	GO:0023052	317	158	4.89 × 10^−46^
Cell proliferation	GO:0008283	170	49	1.10 × 10^−45^
Cell communication	GO:0007154	323	165	1.29 × 10^−45^
Secretion	GO:0046903	118	26	1.90 × 10^−40^
Localization	GO:0051179	306	160	4.32 × 10^−39^
Locomotion	GO:0040011	147	42	8.15 × 10^−39^
Hemopoiesis	GO:0030097	94	17	2.20 × 10^−37^
Homeostatic process	GO:0042592	140	41	2.50 × 10^−36^
Hemostasis	GO:0007599	81	13	1.68 × 10^−35^
Cell differentiation	GO:0030154	225	100	1.15 × 10^−34^
Coagulation	GO:0050817	79	13	5.41 × 10^−34^
Movement of cell or subcellular component	GO:0006928	144	47	1.30 × 10^−32^
Cell adhesion	GO:0007155	115	31	9.89 × 10^−32^
Receptor binding	GO:0005102	119	34	2.07 × 10^−31^
System development	GO:0048731	239	116	2.15 × 10^−31^

P-values were calculated between PPIPs and non-PPIPs using Fisher's Exact Test. For clarity, regulatory and hierarchically related processes were excluded. All PPIP-enriched categories are available in S3 Table.

### Immune and other PPIPs are enriched for viral and bacterial interactions

Many immune genes, even outside the adaptive immune system, are activated by signals from multiple pathogens (e.g. [36, 37]). This 'pathogen pleiotropy' poses an important complication when testing the link between blood parasites and host adaptation, even in genes phenotypically linked to these parasites. To quantify the extent of pathogen pleiotropy in mammals, we compared PPIPs to host proteins known to interact with viruses and bacteria (Methods, VIPs and BIPs). In both cases, we focused only on genes conserved across our focal 24 mammal species. For viruses, we obtained a high-quality list of 1,256 manually curated virus-interacting proteins from [31] (S4 Table). For bacteria, we queried the EBI IntAct database [38] for all deposited interactions between humans and bacteria, which returned 1,250 host proteins (S4 Table).

Overall, we find that 36% of all PPIPs also interact with viruses, 23% with bacteria, and 48% with viruses and/or bacteria—many more than expected by chance (Fig 2C; all p<10^-4^). Unsurprisingly, this overlap is strongest for immune PPIPs (here defined as falling under the GO category *immune system process*), of which 56% interact with multiple pathogens (p<10^-4^). However, nearly 40% of non-immune PIPs also interact with multiple pathogens (p<10^-4^). Some of these "non-immune" proteins may have uncharacterized immune functions, but most are known for their involvement in general cellular processes, including metabolism and signal transduction. This suggests that a diverse array of prokaryotic, eukaryotic, and viral pathogens may interact with a surprisingly small number of host proteins, or alternatively, that these proteins represent a non-specific host response to infection.

### PPIP tissue expression patterns support role in malaria

*Plasmodium* and Piroplasms influence several mammalian tissues as they progress through their complex life cycle. To begin investigating the specificity of PPIPs to malaria-like infections, we examined gene expression in a condensed set of 34 human tissues collected by the GTEx Consortium from uninfected individuals [39] (Methods, Protein Metrics). We first found that PPIPs have an average of 8.2% higher total expression than randomly selected sets of non-PPIPs (p<0.001; S1A Fig). To fairly evaluate PPIP expression enrichment in each tissue, we designed a matched permutation test that compares PPIPs to many, similarly-sized sets of control genes with similar total expression (Methods, Permutation Tests). Throughout this work, we use matched permutation tests to compare PPIPs to many sets of other genes that are controlled for confounding factors. This approach allows us to isolate the evolutionary effects of interactions with *Plasmodium* or Piroplasms from potentially correlated factors, such as high total expression.

**Fig 3:**
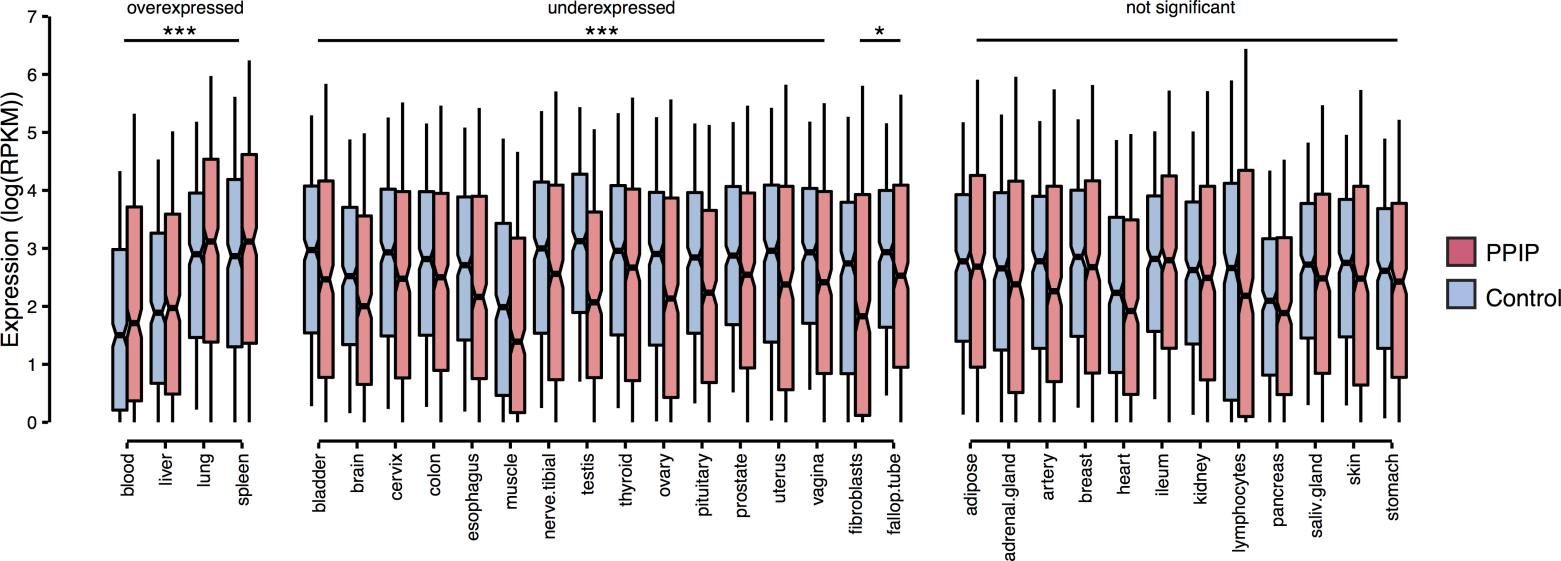
Tissue expression of PPIPs and control genes. The GTEx median RPKM + 1 values for each gene and tissue were log_2_ transformed and plotted for PPIPs (pink) and one set of control genes (blue), matched for total expression. Whiskers denote the range of values 0.1 times the interquartile range from the box. Significance was determined by permutation within each tissue, i.e., by comparing the mean expression of PPIPs to the means of 1000 sets of matched control genes. *** = p<0.001; * = p<0.05.

Compared to matched control proteins, we found that PPIPs were significantly differentially expressed in 20 of 34 tissues, despite the noisiness of this measurement (Fig 3). PPIP expression was underrepresented in 16 tissues, particularly those involved in reproduction, and overrepresented in four tissues: blood, liver, lung, and spleen. All four of these overrepresented tissues are key sites of parasite replication, containment, and/or tissue damage in *Plasmodium* or Piroplasm infections in mammals. This result may be due in part to an ascertainment bias in sampling certain tissues, especially blood, but is also consistent with the biology of host interactions with *Plasmodium* and Piroplasm parasites.

### PPIPs are not like other proteins

We have already shown that PPIPs have three unusual properties—immune enrichment (Table 1), excess interactions with other pathogens (Fig 2C), and high mRNA expression (S1A Fig)—that may influence their rate of evolution. In order to evaluate PPIP adaptation against an appropriate background, we assessed several additional metrics for PPIPs and other proteins in order to control for them in our permutation tests (S5 Table).

First, we examined three additional broad measures of gene function in humans: the density of DNAseI hypersensitive elements; protein expression, as measured by mass spectrometry; and the number of protein-protein interactions (Methods, Protein Metrics). For each of these metrics, PPIPs have significantly higher mean values than sets of random controls, indicating that PPIPs are more broadly functional in humans (S1B–S1D Fig; all p<0.001). We next tested four measures of genomic context that have been linked to the rate of sequence evolution: GC content; aligned protein length; the regional density of protein-coding bases; and the density of highly conserved, vertebrate elements [40–43](Methods). Most of these metrics do not differ between PPIPs and other genes (S1F–S1H Fig), with the exception of conserved element density, which is slightly but significantly lower in PPIPs (mean = 8.0% vs. 8.8%; p = 0.002; S1E Fig).

Based on these results, we expanded our permutation test to match all five of these significantly varying measures of gene function and genomic context while generating sets of non-PPIP control genes. Each non-PPIP was considered an acceptable match for a given PPIP if its values for all five metrics fell within specific ranges of the PPIP values (Methods, Permutation Tests). About 10% of PPIPs were too dissimilar from other proteins to be matched and were excluded from subsequent analysis, but these proteins show similar rates of adaptation to other PPIPs (S6 Table). On average, each retained PPIP could be matched to 34 control genes, allowing the generation of many different sets of ~440 matched controls. This permutation procedure effectively equalized distributions between PPIPs and control genes for all tested functional and evolutionary metrics (compare S1 Fig to S2 Fig).

Finally, one of the largest differences between PPIPs and other proteins is the frequency with which they are discussed in the scientific literature (S1I Fig). The average PPIP has 6.9 times more PubMed citations than the average mammalian protein (Methods, Protein Metrics). This difference was too large to match in the permutation test without excluding the majority of PPIPs. However, we show that the citation frequency of non-PPIPs has no relationship with protein adaptation (p ≥ 0.17; S3 Fig). This indicates that a high rate of citation for PPIPs is not statistically associated with their rate of adaptation.

### PPIPs have experienced elevated rates of adaptation in mammals

**Fig 4:**
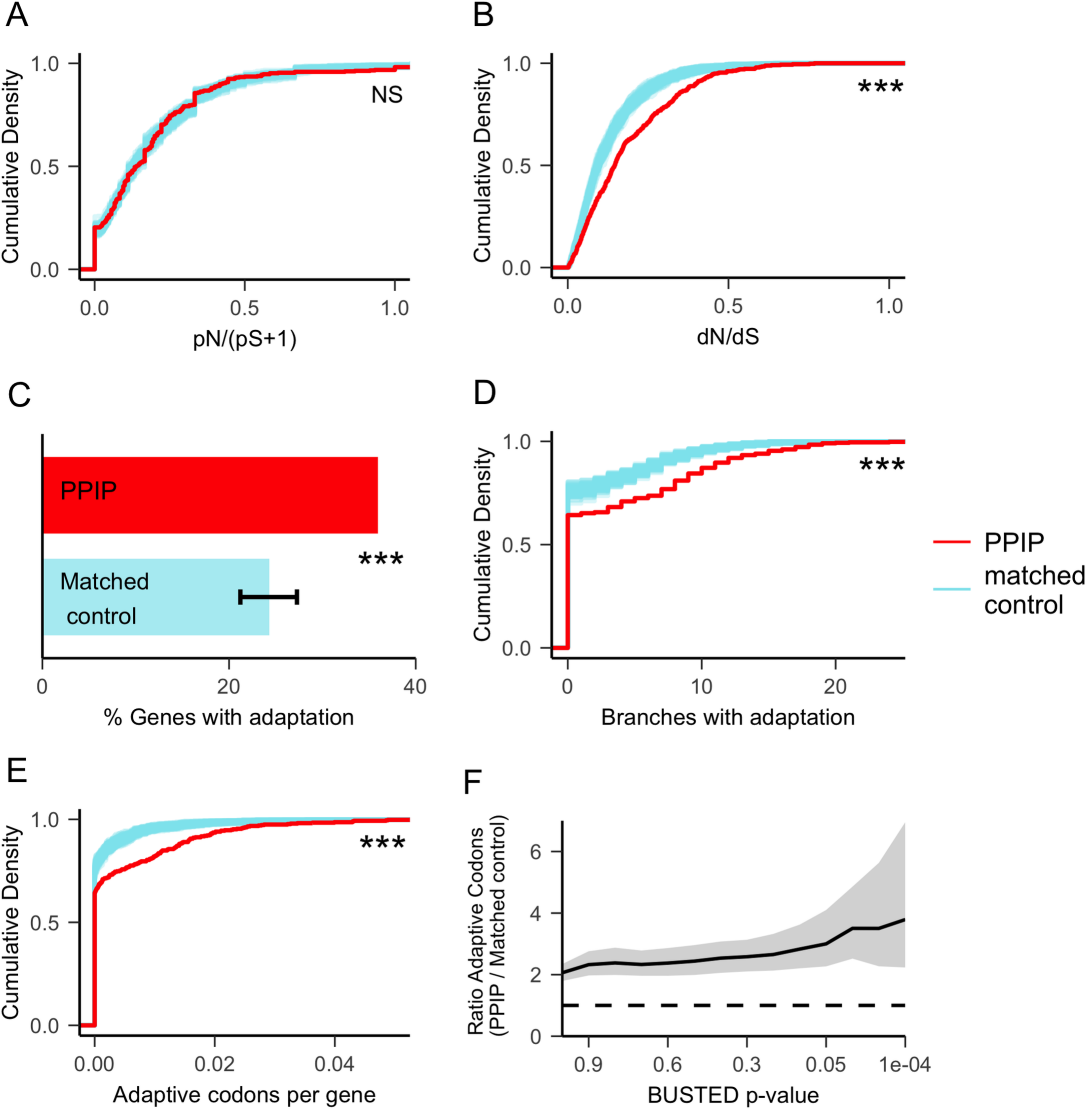
PPIPs have experienced a significant excess of adaptive substitutions in mammals. All comparisons are made between PPIPs and carefully matched control proteins (Methods, Permutation Tests). **(a)** Evolutionary constraint across great apes, as measured by the ratio of non-synonymous to synonymous polymorphisms, is equivalent in PPIPs and matched controls. The thick red line represents the cumulative density of pN/(pS+1) for PPIPs, whereas the cloud of thin blue lines represents the cumulative densities of 100 sets of matched controls. **(b)** Across 24 mammal species, the ratio of non-synonymous to synonymous substitutions is elevated in PPIPs versus matched controls. Lines as in (a). **(c)** BUSTED detects mammalian adaptation in 37% of PPIPs and, on average, 24% of matched controls. Error bars indicate the 95% range of the proportion of matched control genes with evidence of BUSTED adaptation, over 100 sets of matched controls. **(d)** Across the mammalian phylogeny (Fig 1), BS-REL tests identify PPIPs as evolving adaptively on a higher number of branches. Lines as in (a). **(e)** BS-REL tests identify a higher proportion of codons in PPIPs as evolving adaptively. Adaptive codons per gene are given as an average across all branches. Lines as in (a). **(f)** The ratio of adaptive codons in PPIPs versus matched controls increases (p = 7x10^-5^) as the BUSTED threshold for including BS-REL estimates becomes more stringent. The solid line indicates the mean excess; the dashed line indicates the 1:1 expectation; gray shading indicates 95% confidence intervals. In all panels, *** = p<0.001; NS = p>0.05.

After controlling for each metric of function and genomic context (S2 Fig), we asked whether PPIPs exhibit unusual patterns of amino acid substitution and polymorphism in mammals. Importantly, PPIPs have a typical ratio of non-synonymous to synonymous polymorphism in a combined sampling of great ape species (Fig 4A; mean pN/(pS+1) = 0.21 in PPIPs vs. an average of 0.20 in matched controls; p = 0.10; Methods, Protein Metrics). That is, PPIPs do not appear more or less evolutionarily constrained than other similar proteins, bolstering the null expectation that they should evolve at average rates.

In contrast, we find that PPIPs have a significantly elevated ratio of non-synonymous to synonymous substitutions across 24 mammal species (dN/dS = 0.186 in PPIPs vs. an average of 0.128 in matched controls, p<10^−4^). If we make a very conservative (and in many ways unreasonable) assumption that matched controls have experienced no adaptation in the history of mammals, then this 31% excess in dN/dS in PPIPs, despite unremarkable pN/(pS+1), implies that at least 31% of all amino acid substitutions in PPIPs were adaptive. However, given that matched controls have likely experienced at least some adaptation, the proportion of adaptive substitutions in PPIPs is likely to be even larger.

We investigated adaptation in PPIPs in more detail using the BS-REL and BUSTED tests available in the HYPHY software package [44–46] (Methods, Estimating Adaptation). Both tests use maximum likelihood models to estimate the proportion of codons in a protein with dN/dS > 1, consistent with adaptation in some proportion of the protein. BS-REL estimates the exact proportion in each branch of the tree, whereas BUSTED estimates whether it is greater than zero in at least one branch (i.e., whether a gene has experienced positive selection at some point in the history of mammalian evolution).

Both models find additional evidence of excess adaptation in PPIPs. Nearly 36% of PPIPs have BUSTED evidence (at p≤0.05) of adaptation in some part of the mammalian phylogeny, versus 24% of matched controls (p<10^−4^; Fig 4C). PPIPs also have BS-REL evidence for adaptation on more branches of the mammalian tree (p = 1.87× 10^−4^; Fig 4D), as well as for more codons per protein (p<10^−4^; Fig 4E). This excess is robust to the BUSTED p-value threshold used to define adaptation, and increases as the threshold becomes more stringent (Fig 4F, p = 7x10^-5^). PPIPs identified via different kinds of evidence all have more adaptation than expected by chance, although PPIPs that physically interact with parasite proteins have a greater excess of adaptation than PPIPs that change expression during infection (p = 0.032, S4 Fig).

### Pathogen pleiotropy drives adaptation linked to immunity

These matched permutation tests show that PPIPs have experienced an elevated rate of adaptive substitutions in mammals. Although the hundreds of published experiments that define PPIPs (Fig 2A) support the idea that *Plasmodium* and Piroplasms may have driven this adaptation, it remains critical to address PPIP pleiotropy with other pathogens (Fig 2C).

**Fig 5:**
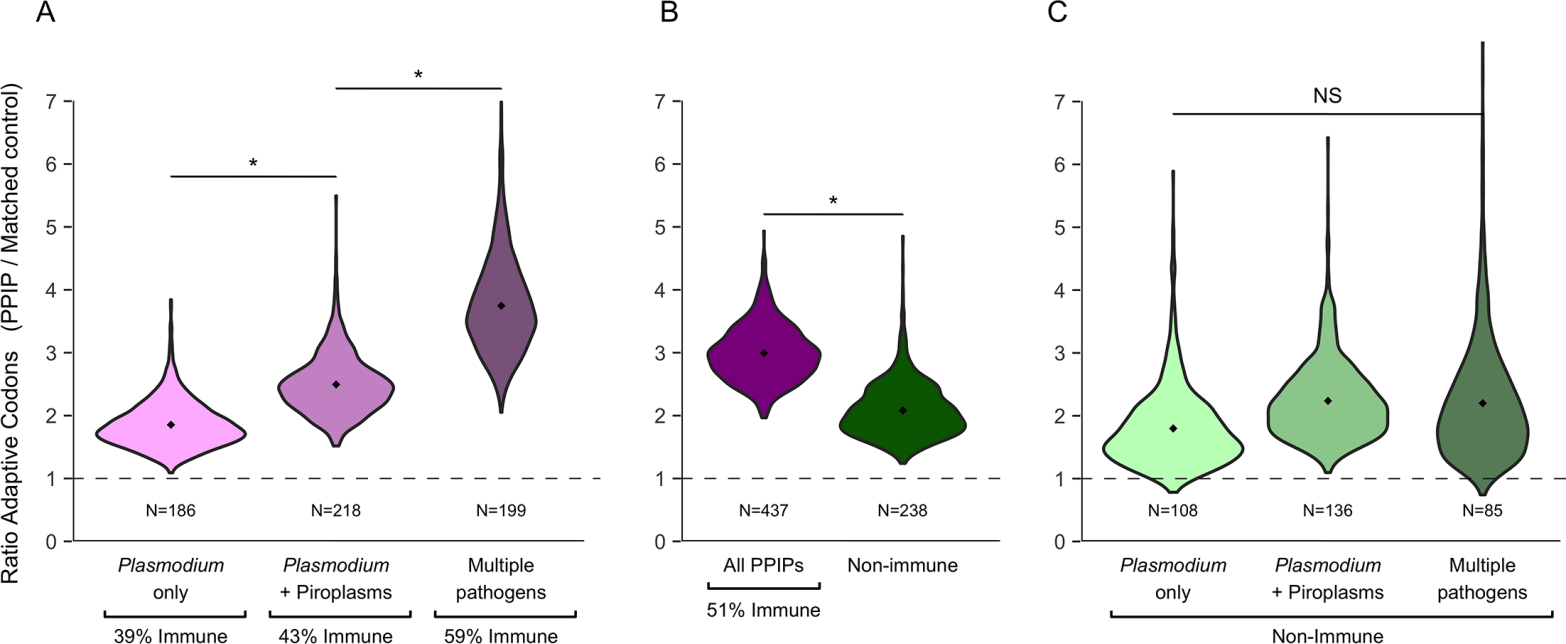
Pathogen pleiotropy is linked to adaptation for immune PPIPs. Each violin plot depicts a set of 1000 ratios comparing the mean proportion of adaptive codons in PPIPs to that in 1000 sets of matched controls. Adaptive codons per gene are an average across all branches. The dashed line indicates the null 1:1 expectation for PPIPs:matched controls. **(a)** PPIPs are divided into those that are only known to interact with *Plasmodium*, those that interact with *Plasmodium* and Piroplasms, and those that also interact with viruses and bacteria. The fraction of PPIPs with annotated immune functions increases with the number of interacting pathogens, as does the ratio of adaptive codons in PPIPs: matched controls. **(b)** All PPIPs compared to PPIPs without annotated immune functions. The ratio of adaptive codons in PPIPs:matched controls is significantly lower for PPIPs without immune functions than for all PPIPs. **(c)** Non-immune PPIPs are divided into those that are only known to interact with *Plasmodium*, those that interact with *Plasmodium* and Piroplasms, and those that also interact with viruses and bacteria. Unlike for all PPIPs (a), interaction with pathogens beyond *Plasmodium* is not significantly correlated with additional adaptation in non-immune PPIPs. In all panels, each violin is significantly above the 1:1 expectation of PPIPs:matched controls (all p≤0.02). Horizontal bars indicate differences between violins; * = p<0.05, NS = not significant.

Based on the available information for many host-pathogen interactions (Methods, VIPs and BIPs), we divided PPIPs into three categories: "*Plasmodium*-only," "*Plasmodium* + Piroplasms," and "multi-pathogen," which includes PPIPs that also interact with viruses and/or bacteria (Fig 2C). For each category, we again matched PPIPs to controls and found significantly more adaptive substitutions than expected (Fig 5A). Among categories, the excess adaptation for PPIPs versus controls is greater when more diverse pathogen interactions are included (p<0.05). That is, *Plasmodium*-only PPIPs have 1.9X more adaptation than expected; PPIPs that interact with *Plasmodium* and/or Piroplasms but are not known to interact with viruses or bacteria have 2.5X more adaptation than expected; and PPIPs that also interact with viruses or bacteria have 3.7X more adaptation than expected (Fig 5A). This result suggests that an increased number and diversity of pathogen interactions drives a cumulative increase in host adaptation.

Importantly, PPIPs that interact with more pathogens are also more likely to have immune functions (Fig 5A, Fig 2C). Only 39% of *Plasmodium*-only PPIPs, versus 59% of multi-pathogen PPIPs, have a GO annotation for *immune system process* (Fig 5A). Immune genes are well known to evolve at rapid rates [47–52]. Here, we also find that non-immune PPIPs have adapted at slower rates than PPIPs as a whole (Fig 5B; p = 0.018; see Methods, Permutation Tests for why immune PPIPs are not analyzed directly). These correlations among immune function, adaptation, and multi-pathogen interactions complicate the link between malaria-like parasites and host adaptation.

Fortunately, these correlations can be disentangled by considering the 239 PPIPs that do not have an annotated immune function. In these non-immune PPIPs, there is a breakdown of the link between adaptation and multi-pathogen interactions (Fig 5C). That is, non-immune PPIPs known to interact only with *Plasmodium* have ~2X more adaptation than expected, and this excess does not significantly increase when PPIPs that interact with additional pathogens are included (all p > 0.17; Fig 5C). We note that this lack of a significant difference is not simply due to reduced power from a reduced sample size, given that subsampling of PPIPs in Fig 5A to the sample sizes in Fig 5C retains complete power to detect differences. Overall, we show that even non-immune PPIPs not known to interact with any pathogens except for *Plasmodium* still show sharply elevated rates of adaptation. Although we lack complete knowledge of host-parasite interactions, to explain this result independently of *Plasmodium* as a selective pressure would require the existence of some other pressure or pathogen, whose interactions with mammalian genes overlap remarkably well with those of *Plasmodium*.

### PPIP adaptation is related to expression in the liver

**Fig 6:**
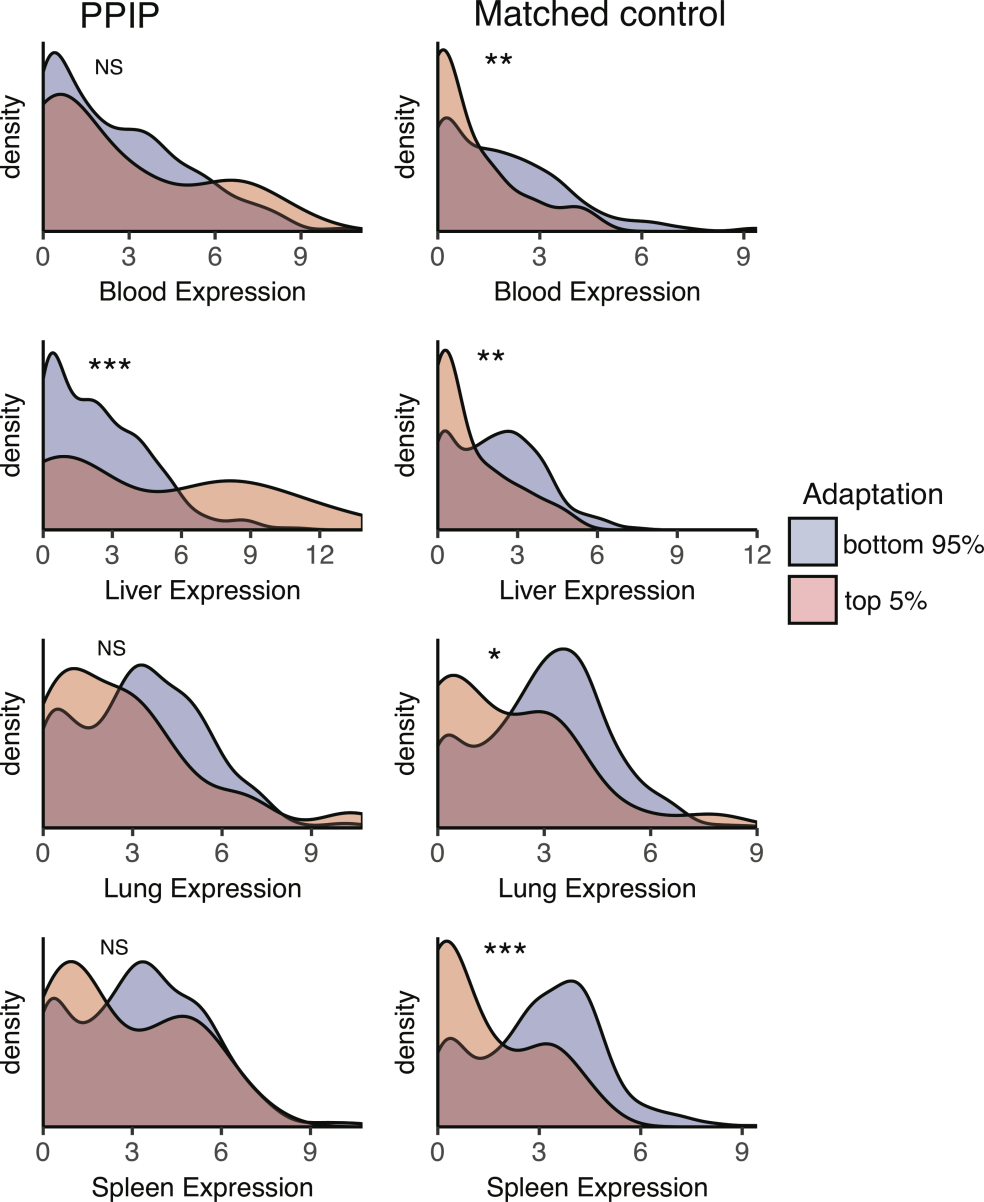
Highly adaptive PPIPs have high expression in the liver. For both PPIPs and controls matched for total expression, the 5% of the gene set with the most adaptive codons (averaged across all branches) was compared to the remainder of the gene set. Expression is plotted as log_2_(GTEx median RPKM + 1) for each set of genes and tissue. For matched controls, highly adaptive genes are expressed at low levels in all malaria-relevant tissues. This pattern differs for PPIPs, particularly in the liver. Significance was determined with the KS test. *** = p<0.001; ** = p <0.01, * = p <0.05; NS = not significant.

Host adaptation to malaria could potentially be concentrated in any malaria-relevant tissue enriched for PPIP expression, specifically blood, liver, lung, and spleen (Fig 3). We used a threshold analysis to test whether expression in these tissues was linked to elevated adaptation. That is, we compared expression patterns for the bulk of genes to patterns in the 5% of genes with the most adaptive codons for both PPIPs and controls matched for total expression (Fig 6).

In sets of matched control non-PPIPs, the most highly adaptive genes are expressed at significantly lower levels than the bulk of control non-PPIPs in all four malaria-relevant tissues (Fig 6). In contrast, for PPIPs, highly adaptive genes are not expressed at significantly lower levels in any of the tissues, despite the same overall sample size and level of total expression. In fact, in the case of the liver, the highly adaptive PPIPs are expressed at significantly *higher* levels than other PPIPs, the opposite direction of the pattern observed in controls (p = 9.8 x 10^−4^; Fig 6). The fact that high expression in the liver is associated with elevated adaptation in PPIPs, but not controls, suggests that the liver may have been a site of particularly strong selective pressures acting specifically on PPIPs.

### PPIP adaptation is widespread throughout mammals

*Plasmodium* and Piroplasm infections have been reported from a wide variety of mammalian species (Fig 1). We tested whether PPIP adaptation is similarly widespread across mammals by applying BUSTED and BS-REL models to subsets of the sequence data within individual mammalian orders (Methods, Order-specific Analyses).

**Fig 7:**
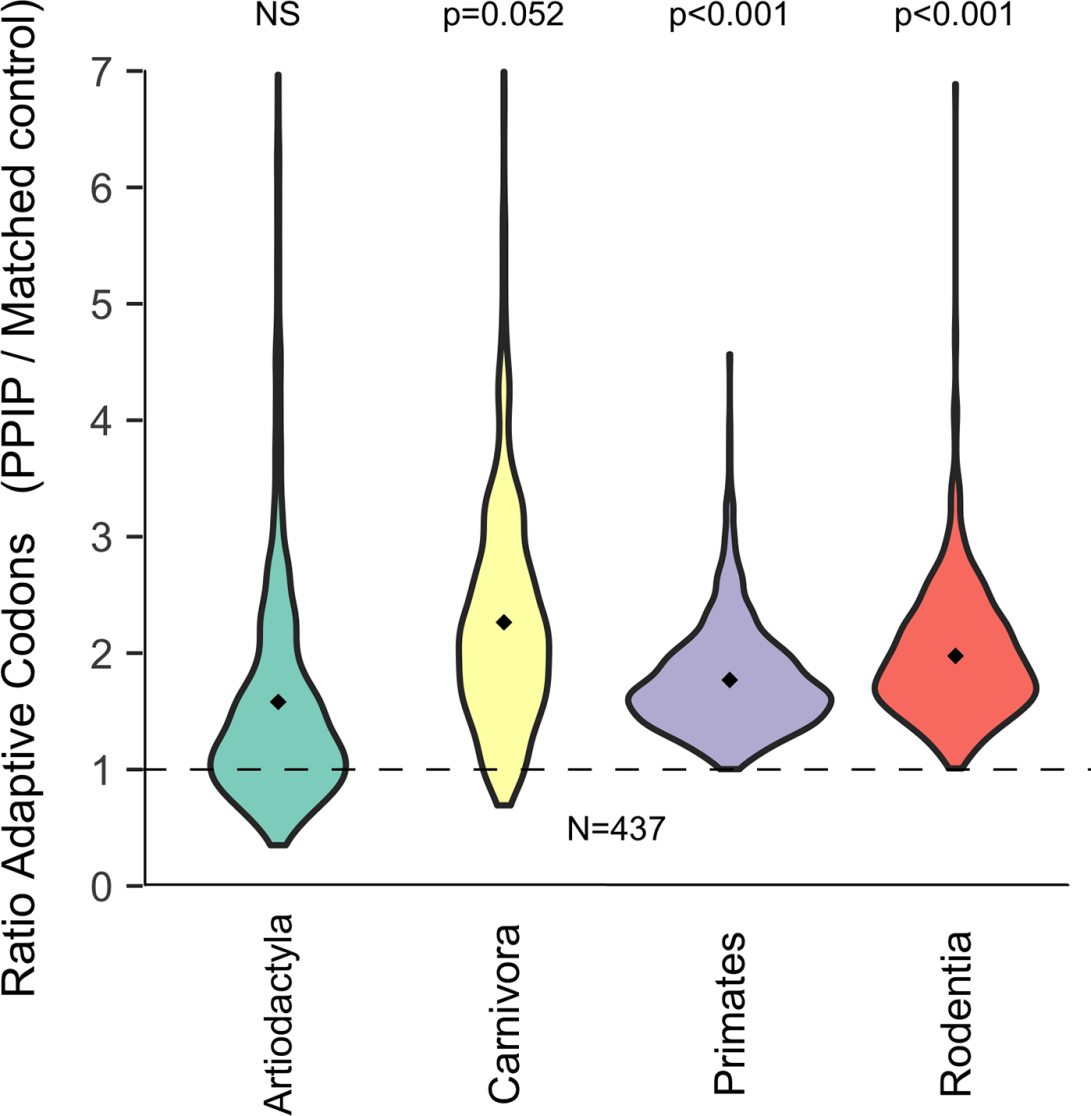
PPIP adaptation is widespread throughout mammals. Each violin plot depicts a set of 1000 ratios comparing the mean proportion of adaptive codons in PPIPs to that in 1000 sets of matched controls. Adaptive codons per gene are an average across all branches contained in the specified clade. The dashed line indicates the null 1:1 expectation for PPIPs:matched controls.

When all PPIPs are considered, we find a highly significant excess of PPIP adaptation in rodents and primates (both p<0.001; Fig 7). Before correcting for multiple testing, the signal is marginally significant in carnivores (p = 0.052) and positive, but not significant, in artiodactyls (p = 0.29). However, it is difficult to compare significance among clades for two reasons. First, we cannot account for differences in evolutionary rate in different groups due to, e.g., generation time. Second, our mammalian tree has not sampled equal numbers of species from each the four clades (Fig 1). We further note that we completely lack statistical power to perform clade-specific analysis on subsets of PPIPs, such as *Plasmodium*- or Piroplasm-only PPIPs (Methods, Permutation Tests). Despite these caveats, clade-specific analyses indicate at least a trend toward high adaptation in PPIPs in all major clades of the mammalian tree.

### Understanding a single case of adaptation to *Plasmodium*

Direct, biochemical interactions between mammalian and parasite proteins may be particularly important drivers of host adaptation (S4 Fig), although such interactions remain uncharacterized for the majority of PPIPs (Fig 2A). We chose one well-studied PPIP to test for a direct relationship, at the amino-acid level, between host adaptation and biochemical host-parasite interactions.

Of the top ten PPIPs with the strongest BUSTED evidence of adaptation, alpha-spectrin (SPTA1) is the only candidate that has been extensively characterized for molecular interactions with *Plasmodium* proteins. Alpha-spectrin is a textbook example of a major structural component of the red blood cell (RBC) membrane. In humans, dozens of polymorphisms in this gene are known to cause deformations of the RBC, which may either be asymptomatic or cause deleterious anemia (reviewed in [53]). These deformations are more common in individuals of African descent, leading to the hypothesis that SPTA1 is involved in malaria resistance in humans.

**Fig 8:**
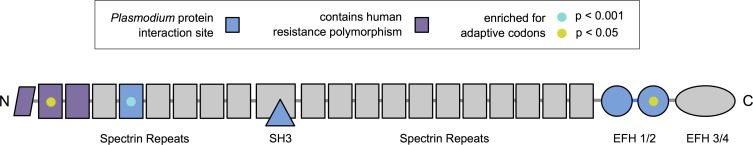
Domains of the alpha-spectrin (SPTA1) protein that interact biochemically with *Plasmodium* proteins are enriched for mammalian adaptation. Adaptation in each codon was determined with MEME on an 85-species alignment of *SPTA1* coding sequence (Methods, Alpha-spectrin). *Plasmodium* interaction sites, and sites of human resistance mutations, were drawn from the literature (see text).

The SPTA1 protein has a well-defined domain structure, and specific interactions with *Plasmodium* proteins are known for three domains (Fig 8). Repeat 4 is the binding site for KAHRP, the major *P*. *falciparum* component of the adhesive 'knobs' that form on the surface of infected RBCs [54]. Another 65-residue fragment containing EF-hand 2 has been shown to bind to PfEMP3, an interaction that destabilizes the RBC skeleton and may allow mature merozoites to egress from the cell [55]. A central SH3 domain can also be cleaved by a promiscuous *Plasmodium* protease called plasmepsin II [56], which mainly functions in hemoglobin digestion [57]. Furthermore, naturally occurring mutations in the first three SPTA1 domains have been shown to impair the growth of *P*. *falciparum* in human RBCs [58–60].

We wished to test whether sites of mammalian adaptation in SPTA1 mapped to any of these *Plasmodium*-relevant domains. To identify adaptive codons with higher precision and power, we aligned *SPTA1* coding sequences from 61 additional mammal species (S7 Table, S10 Table) for analysis in MEME [61] (Methods, Alpha-spectrin). Of the 2,419 codons in this large mammalian alignment, we found that 63 show strong evidence of adaptation (p<0.01), and that these are distributed non-randomly throughout the protein.

Remarkably, three domains—Repeat 1, Repeat 4, and EF-hand 2—are significantly enriched for adaptive codons, after controlling for domain length and conservation (Fig 8; Methods). That is, all three SPTA1 domains with strong evidence of adaptation in mammals are known to either interact specifically with *P*. *falciparum* proteins, or harbor human mutations that provide resistance to *P*. *falciparum*. This overlap is unlikely to occur by chance (p = 0.015) and is robust to the p-value thresholds chosen (S8 Table). Thus, evidence from SPTA1 suggests a specific connection, at least in this well-studied example, between the mechanics of *Plasmodium* infection and adaptation in the host red blood cell.

Notably, we do not claim that all adaptation in SPTA1 is due to pressure from malaria. Adaptation has occurred in at least one codon of SPTA1 on every branch of the 85-species mammalian tree, with the top branches including the base of the Camelidae, *Loxodonta africana* (elephant), *Trichechus manatus* (manatee), and the base of all Eutheria (S11 Table). The especially high density of adaptive changes in camels may be related to the unusual shape of their red blood cells, which has been shown to extend RBC lifespan during chronic dehydration [62, 63]. Nonetheless, when we focus only on the three domains of SPTA1 that are enriched for adaptive substitutions (Fig 8), we find much stronger evidence of adaptation on primate branches than when the entire SPTA1 protein is considered (S11 Table). This observation is consistent with known molecular interactions between *P*. *falciparum* and these specific SPTA1 domains in humans. Together, branch- and domain-specific patterns of adaptation in SPTA1 support malaria as an important, but by no means unique, influence on the evolution of mammalian red blood cells.

## Discussion

In this work, we have identified 490 conserved mammalian proteins that interact with *Plasmodium* or Piroplasm parasites. This large set of PPIPs is a substantial expansion of the list of host proteins traditionally considered to be associated with *Plasmodium* and Piroplasms (e.g. [10]), enabling us to investigate both their broad functional properties and long-term evolutionary patterns.

We find that PPIPs are strongly enriched for immune annotations, although about half are involved in diverse non-immune processes (Table 1). PPIPs are also widely expressed, but particularly overrepresented in the blood, liver, lung, and spleen—tissues highly relevant to the *Plasmodium* and Piroplasm life cycles. We find that PPIPs tend to interact not only with multiple blood parasites (Fig 1B), but also with unrelated bacterial and viral pathogens (Fig 1C). As expected, this multi-pathogen overlap is strongest for immune PPIPs. Somewhat surprisingly, this overlap also extends to non-immune PPIPs, suggesting either that unrelated parasites tend to interact with the same host proteins or that these proteins correspond to some non-specific host response.

Our key result is that PPIPs have been evolving unusually quickly compared to carefully matched non-PPIPs (Fig 4). If we conservatively assume that none of the amino acid substitutions in non-PPIPs have been adaptive, then we may estimate that 31% of amino acid substitutions in mammalian PPIPs have been driven by positive selection. However, because non-PPIPs have also experienced appreciable positive selection (Fig 4C), the true proportion of adaptive substitutions in PPIPs is certainly higher. Regardless of the precise number, it is clear that host proteins that interact with *Plasmodium* or Piroplasm parasites have evolved at an elevated rate, with a substantial proportion of amino acid changes driven by positive selection (Fig 4).

Across mammals, the rate of PPIP evolution is comparable to that of other proteins previously identified as targets of strong positive selection. For example, the antiviral protein PARP14 and the sperm-expressed protein TEX15—which are not PPIPs—represent two classes of proteins that have diversified rapidly in some mammals [64, 65]. When we consider the proportion of codons under positive selection in all our mammalian orthologs, we find that PARP14 ranks in first place (7.2%), TEX15 in third place (5.8%), and the Piroplasm-associated ENTPD1 in second place (6.3%) (S5 Table). As another example, in colobine monkeys, a shift to foregut fermentation is thought to have driven adaptive substitutions in approximately half of the residues of the antibacterial enzyme lysozyme [66]. Across our broader sampling of mammals, lysozyme ranks in 228^th^ place (1.8% adaptive codons), behind 41 PPIPs (S5 Table). It can be difficult to make precise comparisons across studies that include different mammal species, and with the exception of a recent study of viruses [31], few have systematically assessed the importance of particular selective pressures across many mammals. Nonetheless, PPIPs appear to have experienced similar rates of adaptation as some of the better-known mammalian examples.

Given the extreme pleiotropy of PPIPs in regards to other pathogens, a natural question is whether *Plasmodium* and Piroplasms are truly drivers of PPIP adaptation. Our set of 490 PPIPs is large enough to begin parsing the specific effects of blood parasites on mammalian evolution, independent of the effects of other pathogens. When we consider PPIPs that interact only with *Plasmodium* or Piroplasms, but not viruses or bacteria, we find a 2.5X enrichment of adaptation compared to matched controls (Fig 5A). For non-immune PPIPs in particular, additional viral or bacterial interactions do not elevate the excess of adaptation, which remains significantly higher than in matched controls (Fig 5C). This provides some evidence that blood parasites have played a specific role in influencing mammalian protein evolution, both in immune and non-immune genes.

Two additional pieces of evidence are consistent with this idea. First, PPIPs with the highest levels of adaptation are also particularly highly expressed in the liver, opposite to the pattern seen in matched controls (Fig 6). This suggests the possibility that adaptation is related not simply to liver expression, but to parasite interactions that take place in the liver. In *Plasmodium* infections, parasites initially migrate to the liver, invade hepatocytes, and replicate many times before emerging to infect red blood cells [17]. In Piroplasm infections, liver damage is also common and associated with fatality (e.g. [21, 22, 67]). Although some Piroplasms are thought to lack a liver stage [20, 68], a number of studies have reported the presence of *Babesia*, *Rangelia*, or *Cytauxzoon* parasites within the endothelial cells of the liver, among other tissues [23, 69–71]. The liver may thus represent a critical opportunity for PPIPs to ameliorate the effects of *Plasmodium* and Piroplasm infection on the host.

Second, in the well-studied case of alpha-spectrin (SPTA1), we were able to directly investigate the correspondence between sites of host protein adaptation and sites of molecular interactions with *Plasmodium*. We indeed found strong evidence of adaptation in three domains of SPTA1 that are known to participate in molecular interactions with *Plasmodium* parasites (Fig 8), consistent with host-parasite interactions specifically driving mammalian adaptation. Notably, our evidence of adaptation in SPTA1 was derived from long-term evolutionary patterns in dozens of mammal species, whereas molecular interactions with *P*. *falciparum* were identified only in humans. This is analogous to the interspecies evolutionary patterns we observe in the PPIPs identified from intraspecies association studies (S4 Fig). These results suggest that the host cellular machinery underlying extant parasite interactions has been largely conserved over deep evolutionary time, potentially allowing the same proteins to participate in adaptation across multiple time scales.

In the end, despite these three lines of evidence pointing towards *Plasmodium* and Piroplasms specifically driving PPIP adaptation, this conclusion must remain tentative because of our incomplete knowledge of host-pathogen interactions. At one level, many host genes that interact with pathogens likely remain unidentified [31]. At another level, the taxonomic distribution of pathogens on hosts remains quite poorly understood [2]. This is especially problematic for testing whether adaptation in certain mammal lineages corresponds to the densities of specific parasites. For example, based on veterinary records, we may have expected artiodactyls to adapt specifically to Piroplasms but not to *Plasmodium* (Fig 1). However, white-tailed deer in North America were recently discovered to carry a *Plasmodium* species at high frequency [72]. This finding demonstrates that absence of proof is not proof of absence when it comes to the phylogenetic distribution of pathogens, nor to interactions between parasites and host genes. Emerging high-throughput studies of host-pathogen interactions, combined with broader sampling of natural infections, will allow more precise tests of how hosts evolve in response to specific pathogens.

In our case, the fact that non-immune, "*Plasmodium*-only" PPIPs show a clear excess of adaptation (Fig 5) may reflect either specific interactions with *Plasmodium* or incomplete knowledge of interactions with other pathogens. Likewise, high rates of adaptation in PPIPs highly expressed in the liver may reflect adaptation to liver-antagonizing blood parasites, or to other viral and bacterial pathogens that also damage the liver. Our analysis of SPTA1 provides the most compelling evidence of a specific association between adaptation in a PPIP and interactions with *Plasmodium*, but because this is only a single example, we cannot claim that such associations would be found more broadly if other PPIPs were to be studied in similar detail. However, these results are hopeful for our future ability to identify specific selective pressures associated with specific pathogens.

Other future work could also examine PPIPs for evidence of balancing selection, especially as more non-human polymorphism data become available. Several examples of PPIP evolution in humans indicate an important role for the maintenance of polymorphism [11, 73], and it is possible that sampling of PPIP polymorphism in other species has contributed to the elevated divergence shown here. Balancing selection within species and directional selection across species may even be two sides of the same coin, as evidenced by immune and other genes that appear to have experienced both [11, 74] (S5 Table; S4 Fig). Indeed, balanced states have been shown to be a natural consequence of directional selection in fast-changing environments [75]. In human populations with abundant polymorphism data, PPIPs could be used as an important resource for understanding the relationship between these two selective modes.

In conclusion, we show that proteins that interact with *Plasmodium* and Piroplasms comprise a substantial portion of the mammalian proteome; that they exhibit high rates of adaptation across mammals; and that this adaptation may be partially driven by these blood parasites. We hope that the collection of 490 mammalian PPIPs will continue to prove a powerful and continually growing resource for exploring host-parasite interactions and adaptation.

## Methods

### PPIP identification

We queried PubMed for scientific papers containing both a gene name and the term *malaria*, *Plasmodium*, *Babesia*, *Theileria*, *Rangelia*, or *Cytauxzoon* in the title or abstract, as of Feb. 20, 2017. Human gene names were drawn from the HUGO Gene Nomenclature Committee [76] (http://www.genenames.org/) for 9,338 mammalian orthologs (see Methods, Mammalian Orthologs). For each of the genes that returned at least one hit, we manually evaluated the titles of up to 20 associated papers to assess the link between the gene and a malaria phenotype. Many acronyms used to represent genes are also used as abbreviations for techniques, locations, drugs, or other phrases. Consequently, most genes could be eliminated based on their nominal connection with papers addressing non-genetic aspects of malaria.

For papers discussing genes, we examined the abstracts for the presence and type of evidence connecting genes to malaria phenotypes. In cases where the abstract was ambiguous, we examined the full text of the paper.

To limit the number of false positives, we did not classify PPIPs using evidence from RNAseq or other high-throughput experiments. Gene expression is typically regulated via large, interconnected networks (e.g. [35]), such that high-throughput experiments can identify hundreds or thousands of genes whose expression is perturbed by infection. Many of these differentially expressed genes may have very small or indirect impacts on the progression of malaria, making them unlikely to be important targets of malaria-related selection. In contrast, low-throughput expression experiments are typically based on *a priori* knowledge or hypotheses of the more direct roles of a few host genes in malaria.

Focusing on candidate genes may inflate the rate of false positives in genetic association studies [77]. Here, we make substantial efforts to ensure that any bias potentially related to PPIP identification, such as the popularity of a gene, does not impact our results (S1–S3 Figs). While we cannot guarantee that every PPIP is a true positive, in part because replication has often not been attempted, PPIPs as a whole do appear to represent a meaningful class of genes. In general, misclassification of either PPIPs or non-PPIPs for any reason (false negatives or false positives) would reduce any true difference between the two categories, weakening our results.

### Mammalian orthologs

We used BLAT to identify homologs of 22,074 human coding sequences in 24 high-depth mammal genomes (Fig 1). We retained orthologs which (1) had best reciprocal hits in all 24 mammal species, (2) lacked any in-frame stop codons, (3) were at least 30% of the length of the human sequence, and (4) had clearly conserved synteny in at least 18 non-human species. Coding sequences for the resulting 9,338 proteins were aligned with PRANK (S1 File), and any codon present in fewer than eight species was excluded from analysis. Additional details are available in [31].

### Protein metrics

GO annotations were downloaded in October, 2015 from the Gene Ontology website [78] (http://geneontology.org/). Tests of enrichment were performed using Fisher's Exact Test.

Expression data for 53 human tissues were downloaded from the GTEx portal (http://www.gtexportal.org/home/) on March 6, 2017. These 53 tissues were condensed into a set of 34 tissues by averaging the RPKM across multiple tissue samples from adipose, aorta, artery, brain, cervix, colon, esophagus, heart, and skin. Each data point was then transformed as *log*_*2*_*(RPKM+1)*. Total expression for each gene was calculated as the sum of these transformed values across all 34 tissues.

Regions of DNaseI hypersensitivity, combined from 95 cell types, were obtained from the databases of the ENCODE Project Consortium [79] (https://www.encodeproject.org/). The density of DNaseI hypersensitivity regions was calculated in 50 Kb windows centered on each ortholog.

Protein expression levels were obtained from the Human Proteome Map [80] (http://www.humanproteomemap.org/), which used high resolution and high accuracy Fourier transform mass spectrometry experiments. We summed spectral values over 30 tissues and cell types and took the log of these total values.

The log number of interacting partners for each human protein was obtained from the Biogrid Database [81] (http://thebiogrid.org/), curated by [82].

Genomic elements conserved in 46 vertebrate species, derived from PhastCons [43], were downloaded from the UCSC genome browser (http://hgdownload.cse.ucsc.edu/goldenPath/hg19/phastCons46way/). Conserved element density was calculated within 50 kb windows centered on each gene in the human reference. Coding density was calculated from coding nucleotides in the same 50 Kb windows. The length and GC content of each protein was derived from the mammalian alignment.

The citation frequency of each gene was determined by the number of citations linked to its PubMed Gene page (http://www.ncbi.nlm.nih.gov/gene) as of May 11, 2017.

Polymorphism data for great apes—chimpanzee, gorilla, and orangutan—was obtained from the Great Apes Genome Project [83]. For each individual species, the counts of polymorphic sites are low, making the pN/pS ratio a noisy measure. This problem was alleviated by combining all the great ape data, which provided an overall control for the level of purifying selection across multiple species.

### VIPs and BIPs

Virus-interacting proteins (VIPs) were manually curated in [31] in the same manner as PPIPs. To our knowledge, no similar collection of high-quality interactions is available for other pathogens. Therefore, we queried the EBI IntAct database (http://www.ebi.ac.uk/intact/) for protein interactions between Kingdom Bacteria (taxid:2) or Phylum Apicomplexa (taxid:5794) and humans (taxid:9606). This approach, while much faster than manual curation, is less ideal for two reasons: (1) many interactions are not included in the database (e.g., only 17 human-*Plasmodium* interactions are included in IntAct), and (2) many of the included interactions are based on high-throughput assays, including yeast two-hybrid experiments, which suffer from both false negatives and false positives [84]. Consequently, we do not perform rigorous analysis specifically for bacterial-interacting proteins, as has been done for PPIPs and VIPs [31]. Rather, we use IntAct data on bacterial interactions only to classify PPIPs as 'multi-pathogen' or not (Fig 5).

### Permutation tests

PPIPs were compared to other sets of genes using a permutation test of the mean. That is, the mean value for PPIPs was compared to the mean value of many sets (1,000–10,000) of control genes. P-values were defined as the fraction of permutations where the control mean was more extreme (usually, higher than) than the PPIP mean.

PPIPs differ from other genes in a number of ways (S1 Fig). In order to evaluate PPIPs against a fair background, sets of control genes were selected that were matched to PPIPs by important functional and evolutionary metrics (S2 Fig). This matched permutation approach allows the evolutionary effects of interacting with *Plasmodium* or Piroplasms to be isolated from correlated factors that may also influence evolutionary rate.

For the analyses shown in Figs 4, 5 and 7, each PPIP was matched to a set of control proteins based on similarity in five metrics: mRNA expression, protein expression, protein-protein interactions, DNaseI density, and conserved element density. A control protein was considered a PPIP 'match' if each of its five values fell within a given range, based on the PPIP values (S9 Table). For example, margins of min = 0.1 and max = 0.2 for mRNA expression would mean that, for a control protein to be matched to a PPIP, the mRNA expression of the control must fall between 90–120% of the mRNA expression of the PPIP. The goal was to maximize the number of matched controls per PPIP while creating control sets that were statistically indistinguishable from PPIPs for all five metrics (S2 Fig). To achieve this balance, maximum margins were iteratively chosen that yielded average p-values for all metrics of at least 0.1 over 100 permutations. Once appropriate margins were found, matched control sets of equal size to the PPIP set were obtained by randomly sampling one matched control protein for each PPIP.

Margins for the main permutation test (Fig 4) are given in S9 Table. For subsets of PPIPs (e.g. Fig 5A), the margins were altered to generate well-matched controls in every case. About 90% of PPIPs were typically matched and the rest excluded. Sets of matched controls were chosen based on the distribution of PPIP values included in each test, so whether any given PPIP was matched depended on the other PPIPs in the test (i.e., one extreme PPIP may or may not be balanced out by another). Therefore, the sum of matched PPIPs across categories differs slightly from the total.

Notably, the pool of immune controls is relatively small (966 genes) compared to the pool of non-immune controls (7,548 genes)(S3 Table). This made it difficult to match immune PPIPs to immune controls without discarding many immune PPIPs. Consequently, to test hypotheses of faster immune adaptation, we compared all PPIPs to all controls and non-immune PPIPs to non-immune controls (Fig 5).

### Estimating adaptation

The codeml model m8 from the PAML package [85] was used to estimate dN/dS for each gene across 24 mammal species (Fig 4B). However, branch-site tests in PAML rely on assumptions that may be violated in the case of recurrent adaptation to a pervasive selective pressure (see [31]). Consequently, we also implemented maximum-likelihood branch-site tests in the better-performing HYPHY package [44]. The BUSTED algorithm [45] was used to detect overall evidence of positive selection at any branch in the mammalian tree, and BS-REL was used to estimate the proportion of positively selected codons in each gene on each branch. Both of these algorithms rely on the same underlying codon model; details of the model are described in [44, 45] and reviewed in [31]. Unless otherwise specified (i.e., Fig 4F), codons identified by BS-REL were 'counted' as adaptive if the BUSTED p-value for that gene was ≤0.05.

We note that we did not employ the classical McDonald-Kreitman (MK) test to test for adaptation across multiple branches of the mammalian tree. The MK test estimates the proportion of adaptive substitutions in a protein for a single lineage, based on polymorphism within that lineage and comparison to an outgroup [86]. Here, our questions concern the evolution of proteins in multiple lineages, many of which lack polymorphism data. The methods in HYPHY are designed to simultaneously test for adaptation in multiple lineages, explicitly within the context of their phylogenetic relationships, based on a single sequence from each species. Therefore, BS-REL, BUSTED, and MEME are more powerful and appropriate for our data and questions than the MK test.

### Order-specific analyses

We split the mammal-wide alignments for each gene into four non-overlapping alignments corresponding to the following clades: **primates** (human, chimpanzee, gorilla, orangutan, gibbon, macaque, baboon, marmoset, bush baby), **rodents** (mouse, rat, guinea pig, squirrel, rabbit), **carnivores** (panda, ferret, dog, cat), and **artiodactyls** (sheep, cow, pig). We excluded microbat, elephant, and horse, as these species are not closely related to any of the four major groups [15] (Fig 1). However, we included rabbit with rodents because they are more closely related. We ran BUSTED on each alignment to yield a p-value of clade-specific adaptation for each gene.

PPIPs were matched to controls as described above (Methods, Permutation Tests). However, rather than counting BS-REL adaptive codons in all branches if the tree-wide BUSTED p≤0.05, we (1) kept each clade codon count separate, (2) counted codons only on branches within a clade, and (3) counted codons only if the clade-specific BUSTED p≤0.05.

An attempt was made to examine clade-specific adaptation in *Plasmodium*-only and Piroplasm PPIPs separately. However, down-sampling PPIPs to the numbers actually present in each subset resulted in dramatically increased variance in the estimates of adaptation, which eliminated statistical power to distinguish between PPIPs and controls.

### Alpha-spectrin

Alpha-spectrin homologs were initially identified in 88 mammal species using NCBI Gene (http://www.ncbi.nlm.nih.gov/gene/?Term=ortholog_gene_6708). The sequence of the longest mRNA transcript for each species was downloaded using E-Utilities, and each transcript was trimmed to the longest ORF using TransDecoder [87] (http://transdecoder.github.io/). Coding sequences with <50% of the human CDS length were removed. The remaining 85 coding sequences were aligned with PRANK [88] using default settings (S7 Table). The alignment was manually inspected and corrected using JalView [89]. A phylogenetic tree for the 85 species was also obtained from phyloT (http://phylot.biobyte.de/) using NCBI Taxonomy (S10 Table).

To analyze positive selection in specific domains of alpha-spectrin, we employed the HyPhy test MEME [61] rather than BS-REL. For a given gene, BS-REL estimates a proportion of codons under positive selection on each branch of a phylogeny, but does not specifically identify the adaptive codons. In contrast, MEME tests each individual codon for positive selection across all branches. MEME also estimates a probability of adaptation for that codon on each branch. Unlike BS-REL, then, MEME is capable of identifying specific codons that have evolved adaptively.

We used the domain designations from SMART [90] (http://smart.embl-heidelberg.de/) to assign 92.2% of *SPTA1* codons to one of 25 domains (S8 Table). Then, for each domain, we calculated an 'adaptation score' as:

a/v

where *a* measures adaptation (the proportion of codons within the domain with MEME p≤0.01*) and *v* measures variability (the proportion of codons within the domain that vary among species, i.e., are not 100% conserved). This score controls for domain length as well as the presence of invariable sites, as both components represent proportions of codons within the domain. To calculate the significance of each domain's adaptation score (i.e., to ask, is it higher than expected?), we randomly permuted codons among domains 10,000 times.

*We also tested MEME p-value cutoffs of 0.1, 0.5, 0.005, and 0.001 for defining *a*; these results are available in S8 Table. The results for p≤0.01, which are reported in the main text, are representative across these cutoffs.

## Supporting information

S1 Fig PPIPs differ from random control genes by several functional and evolutionary metrics. Cumulative distributions show the fraction of a given gene set on the y-axis with values less than or equal to the value on the x-axis. The cumulative distribution for PPIPs is shown as a thick red line, while the 100 sets of controls are shown as a cloud of thin blue lines. If the red line appears below the blue cloud, it indicates that the PPIP distribution is shifted toward higher values. *** = p<0.001, ** = p<0.01, NS = p>0.05.
(TIFF)

S2 Fig The matched permutation procedure effectively equalizes PPIPs and control proteins on all tested functional and evolutionary metrics. Lines and markings as in S1 Fig.
(TIFF)

S3 Fig Adaptation is not related to citation frequency. **(a)** A linear regression (dashed line) between citation number and adaptive codons for all mammalian orthologs (both PPIP and non-PPIP) shows no relationship between these variables. Adaptive codons per gene are an average across all branches. **(b)** The top 20% most highly cited non-PPIPs do not differ from random non-PPIPs in their cumulative distribution of adaptive codons, averaged for each gene across all branches.
(TIFF)

S4 Fig All PPIP evidence types identify sets of genes with a significant excess of adaptation. Each violin plot depicts a set of 1000 ratios comparing the mean proportion of adaptive codons in PPIPs to that in 1000 sets of matched controls. Adaptive codons per gene are an average across all branches. Each violin is significantly above the 1:1 expectation of PPIPs:matched controls (all p<0.001). PPIPs identified by biochemical interaction evidence have significantly more adaptation than those identified by expression evidence (p = 0.032, permutation test), but comparisons between all other groups are not significant (p>0.05).
(TIFF)

S1 Table Evidence for *Plasmodium* and Piroplasmid infection in mammals. Because many sequenced species have not been thoroughly tested for blood parasites, evidence from related species is used as noted.
(XLSX)

S2 Table Mammalian PPIPs linked to *Plasmodium* through literature abstracts. The gene name, host species, parasite species, study IDs, and evidence type are given for each PPIP. Note that some well-known malaria-related genes (such as the glycophorin family) are not included because they are not sufficiently conserved among mammals (Methods, Mammalian Orthologs).
(CSV)

S3 Table All GO categories enriched for PPIPs. (XLSX)

S4 Table Ensembl IDs of virus- and bacteria-interacting mammalian proteins. See Methods, VIPs and BIPs for data sources.
(XLSX)

S5 Table Functional and evolutionary metrics for PPIPs and non-PPIPs used in the permutation tests. 164 of the 9,338 mammalian orthologs lacked data for one or more metrics and were excluded. See Methods, Protein Metrics for data sources.
(CSV)

S6 Table PPIPs that had no matches in the main permutation test (Fig 4) and were excluded from further analysis. See Methods, Protein Metrics for data sources.
(CSV)

S7 Table Alignment of SPTA1 sequences from 85 mammal species. (FA)

S8 Table Alpha-spectrin domain analysis. The boundaries of the protein domains were defined with SMART (Methods, Alpha-spectrin). Columns E-I contain p-values for the enrichment of each domain for adaptive codons, when adaptive codons are defined by the MEME p-value threshold in the heading.
(XLSX)

S9 Table Margins used to match control genes to PPIPs in the main permutation test (Fig 4). Each margin represents a percentage of the PPIP value, so that 'min_margin' and 'max_margin' define a range around the PPIP value into which, to be acceptable for matching, a control value must fall.
(XLSX)

S10 Table Phylogeny of 85 mammalian species used in the MEME analysis of SPTA1. (NWK)

S11 Table Distribution of adaptive codons on branches of the 85-species mammalian tree. The number of adaptive codons is given per branch for both the entire SPTA1 protein and for only the domains enriched for mammalian adaptation (Fig 8).
(XLSX)

S1 File Protein alignments for 9,338 mammalian orthologs. (ZIP)
