# Supplementary figures and images for: High rate of adaptation of mammalian proteins that interact with Plasmodium and related parasites
Source: PLoS Genet. 2017 Sep 28;13(9):e1007023. doi: 10.1371/journal.pgen.1007023 (PMC5634635; doi:10.1371/journal.pgen.1007023)

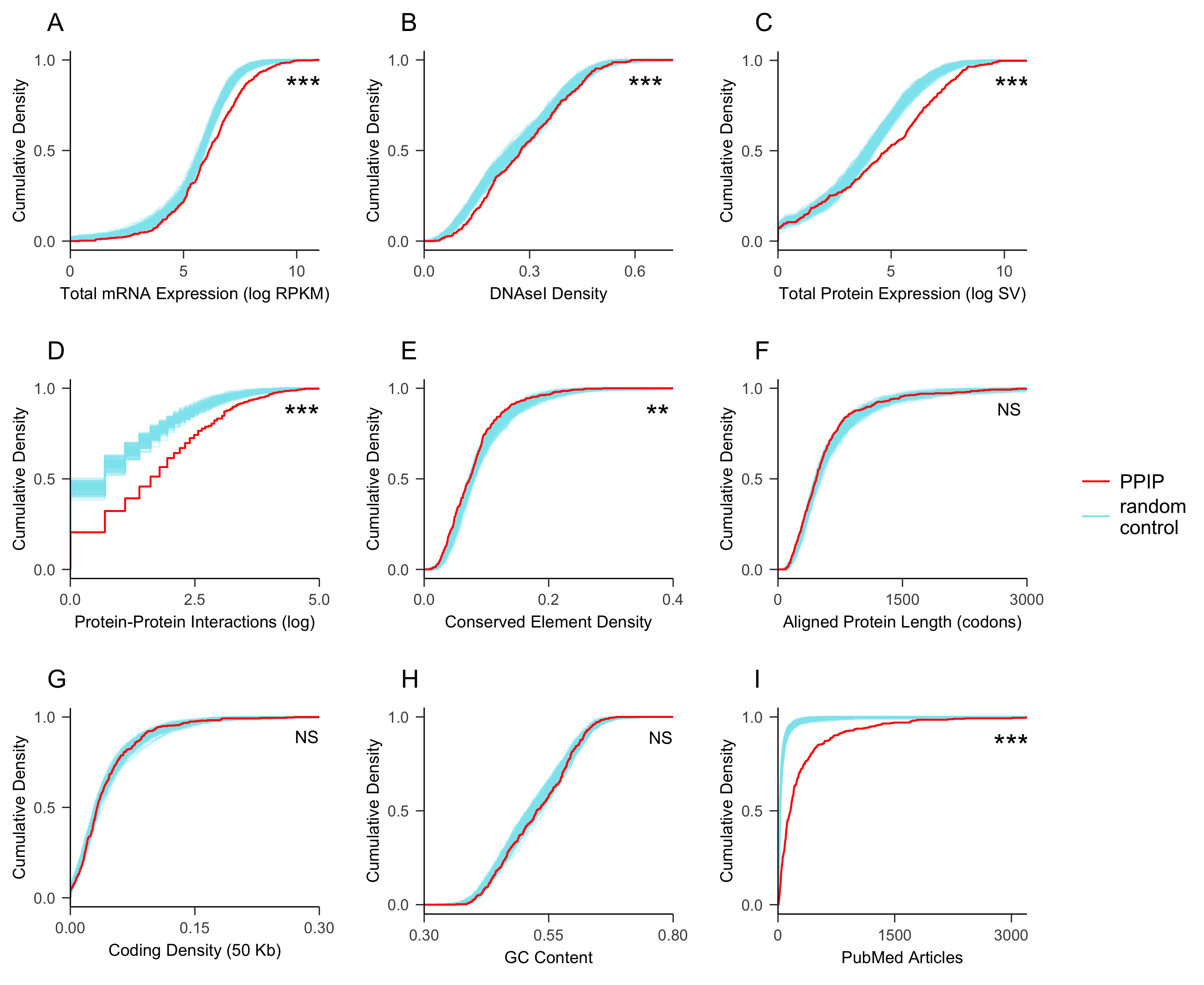

Supplement: S1 Fig — Cumulative distributions show the fraction of a given gene set on the y-axis with values less than or equal to the value on the x-axis. The cumulative distribution for PPIPs is shown as a thick red line, while the 100 sets of controls are shown as a cloud of thin blue lines. If the red line appears below the blue cloud, it indicates that the PPIP distribution is shifted toward higher values. *** = p<0.001, ** = p<0.01, NS = p>0.05. (TIFF) [file pgen.1007023.s001.tiff]

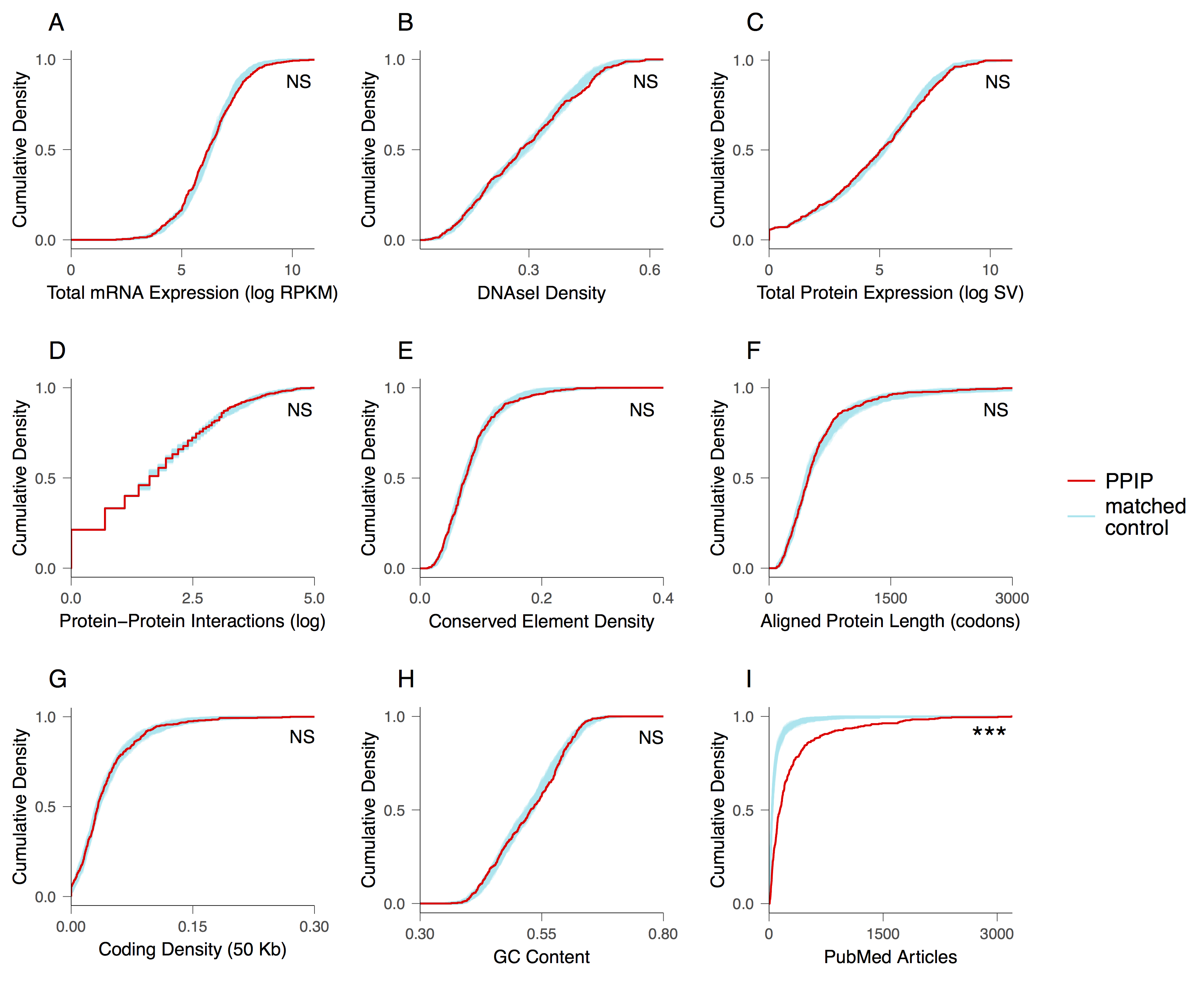

Supplement: S2 Fig — Lines and markings as in S1 Fig. (TIFF) [file pgen.1007023.s002.tiff]

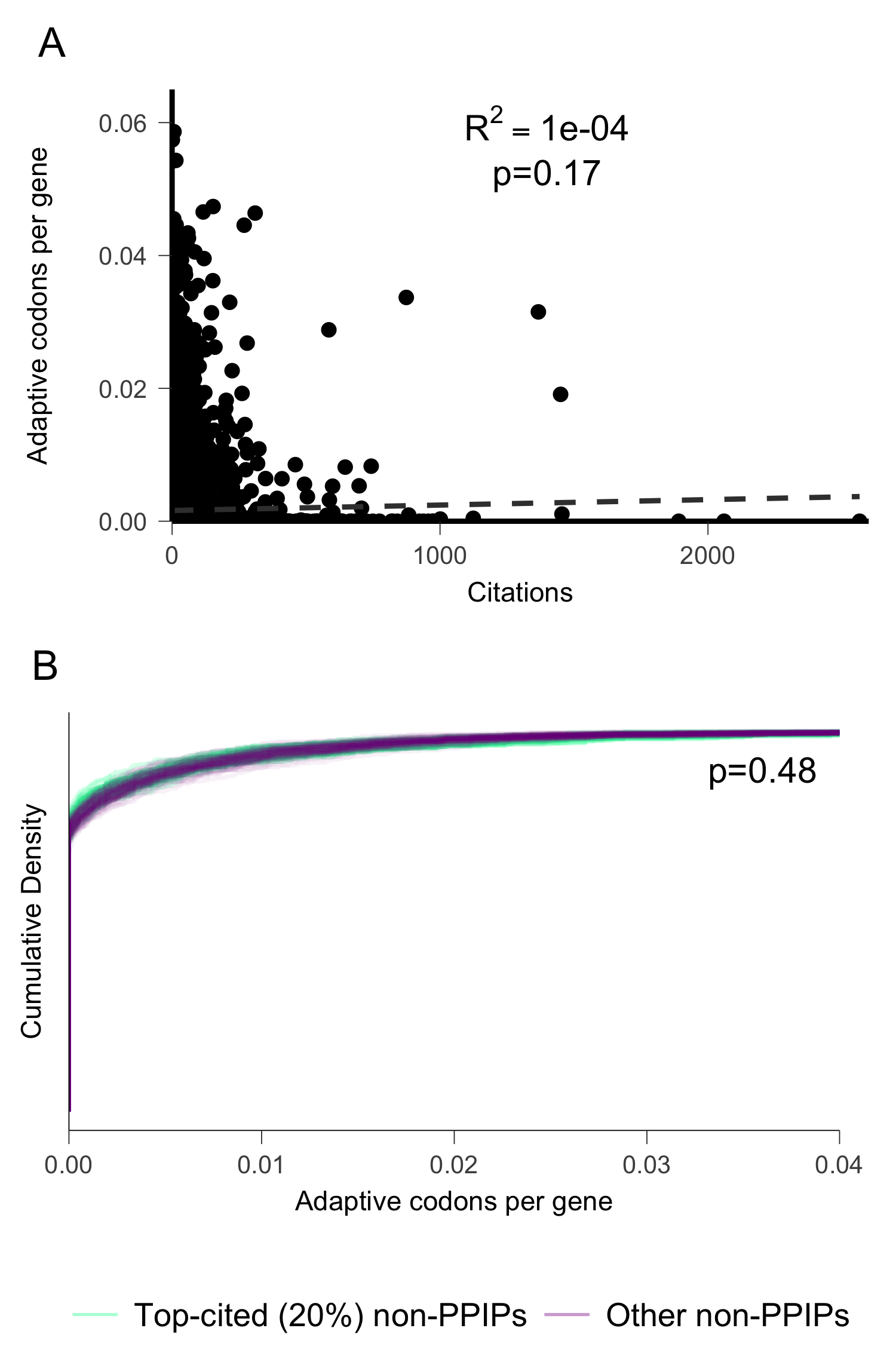

Supplement: S3 Fig — (a) A linear regression (dashed line) between citation number and adaptive codons for all mammalian orthologs (both PPIP and non-PPIP) shows no relationship between these variables. Adaptive codons per gene are an average across all branches. (b) The top 20% most highly cited non-PPIPs do not differ from random non-PPIPs in their cumulative distribution of adaptive codons, averaged for each gene across all branches. (TIFF) [file pgen.1007023.s003.tiff]

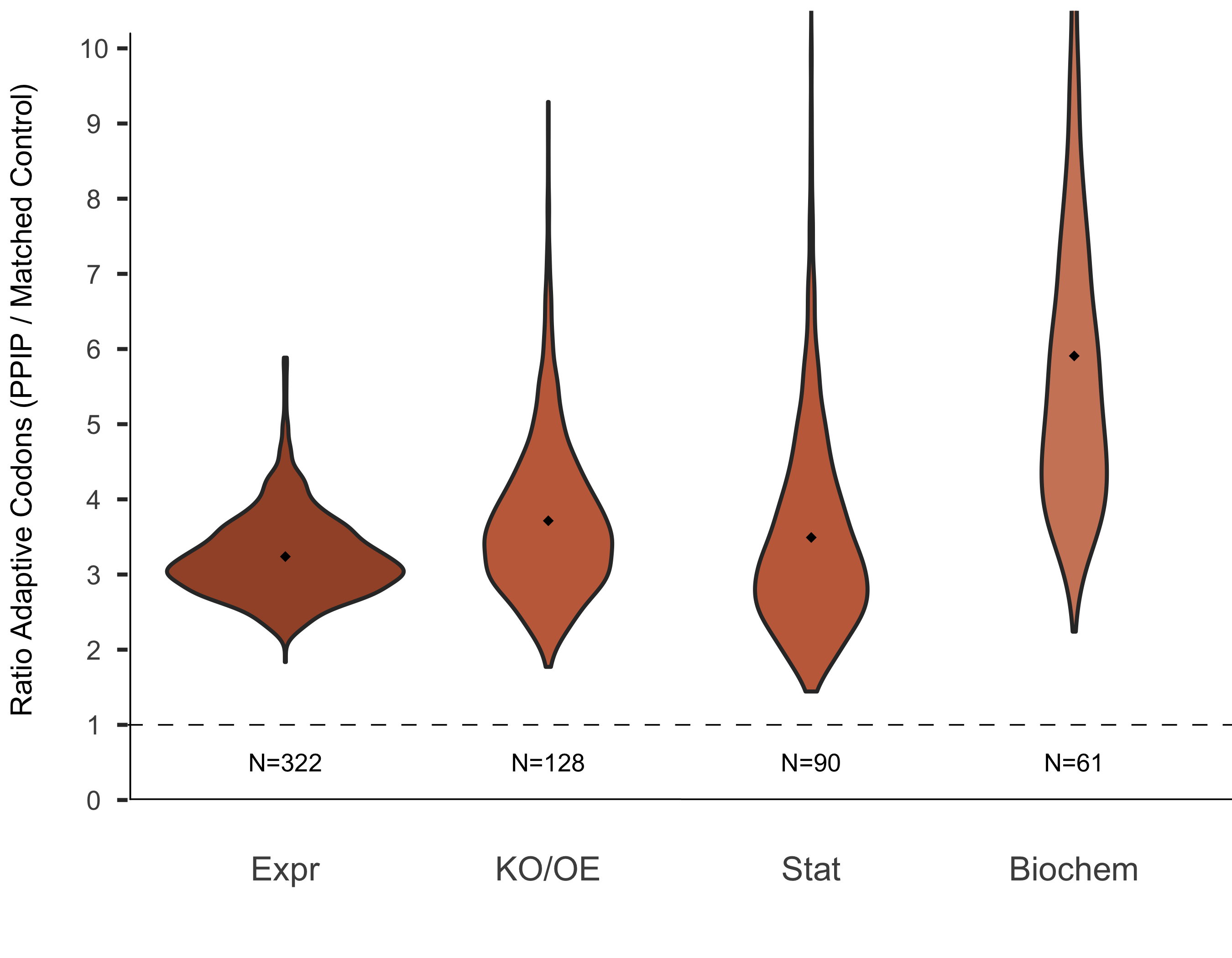

Supplement: S4 Fig — Each violin plot depicts a set of 1000 ratios comparing the mean proportion of adaptive codons in PPIPs to that in 1000 sets of matched controls. Adaptive codons per gene are an average across all branches. Each violin is significantly above the 1:1 expectation of PPIPs:matched controls (all p<0.001). PPIPs identified by biochemical interaction evidence have significantly more adaptation than those identified by expression evidence (p = 0.032, permutation test), but comparisons between all other groups are not significant (p>0.05). (TIFF) [file pgen.1007023.s004.tiff]
